# Bilateral Temporomandibular Joint Ankylosis Treated With Combined Intraoral and Extraoral Gap Arthroplasty: A Case Report

**DOI:** 10.7759/cureus.112895

**Published:** 2026-07-18

**Authors:** Kiyosato Hino, Shogo Kikuta, Shinichiro Terasaki, Jingo Kusukawa

**Affiliations:** 1 Oral and Maxillofacial Surgery, Social Insurance Tagawa Hospital, Tagawa, JPN; 2 Dental and Oral Medical Center, Kurume University School of Medicine, Kurume, JPN

**Keywords:** extraoral approach, gap arthroplasty, intraoral approach, post-traumatic ankylosis, tmj ankylosis

## Abstract

Temporomandibular joint (TMJ) ankylosis causes severe limitation of mouth opening and functional impairment. Surgical management requires careful selection of approach and interpositional material to prevent recurrence and preserve function. We report a case of post-traumatic bilateral TMJ ankylosis treated by gap arthroplasty using different surgical approaches on each side. Because the patient had a right ocular prosthesis, preservation of the left facial nerve and visual function was considered essential. Therefore, the right side was managed using an extraoral approach with a temporalis muscle flap, whereas the left side was treated intraorally using a buccal fat pad flap. Postoperative rehabilitation resulted in improved mouth opening and stable occlusion without clinical evidence of recurrence during the 3.5 years of follow-up. This case highlights the importance of individualized surgical planning based on anatomical and functional considerations in the management of bilateral TMJ ankylosis.

## Introduction

Temporomandibular joint (TMJ) ankylosis restricts mouth opening and impairs mastication, markedly reducing quality of life (QOL) [1]. Traumatic TMJ ankylosis, particularly following bilateral condylar fractures, presents a complex therapeutic challenge. Without appropriate treatment, it may lead to progressive joint dysfunction and jaw deformity [2].

Gap arthroplasty (GA) is a common surgical strategy that restores movement by removing the ankylosed segment and creating an interarticular gap [3]. The extraoral approach provides wide access but risks facial nerve injury and scarring [4]. The intraoral approach is more aesthetic but has limited visibility and complexity [5].

We report a rare case of post-traumatic bilateral TMJ ankylosis treated with GA using an extraoral approach on the right side and an intraoral approach on the left side. This case highlights the importance of individualized surgical planning based on patient-specific anatomical and functional considerations in the management of complex TMJ ankylosis.

## Case presentation

A 47-year-old male patient with no notable family history underwent right eye enucleation at age 10 due to trauma. In 2019, he sustained bilateral condylar fractures and a right mandibular body fracture in a car accident. Open reduction and internal fixation were performed for the body fracture, while condylar fractures were treated conservatively. Despite initial exercises, mouth opening declined after transfer to another hospital. By July 2020, the interincisal distance was 6 mm (Figure 1).

**Figure 1:**
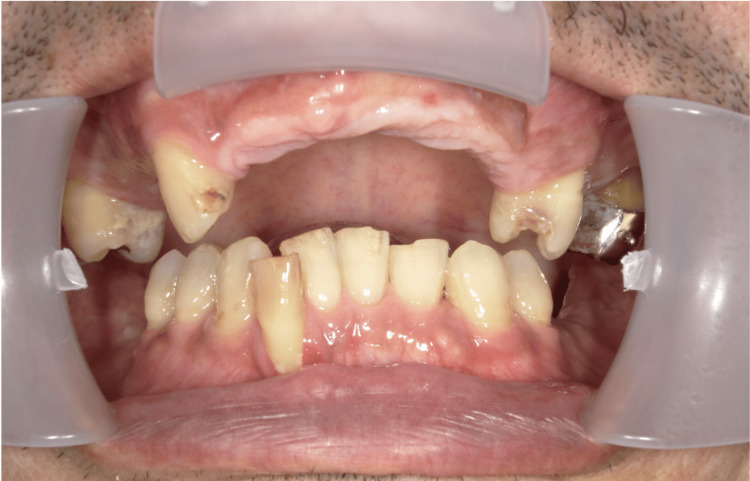
Intraoral photographs obtained at the time of admission to our center. Temporomandibular joint movement was not palpable, and pain was observed in both temporomandibular joint areas during mouth opening and closing. Intraoral examination revealed limited mouth opening, with an intercanine distance of 6 mm on the right side.

Cone-beam computed tomography imaging revealed bony ankylosis of both joints (Figure 2).

**Figure 2:**
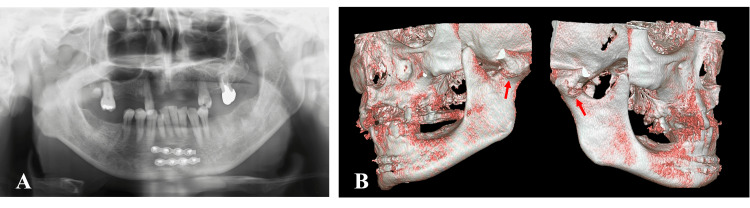
Preoperative panoramic X-ray images and cone-beam computed tomography 3D reconstruction images. (A) The panoramic X-ray image shows adhesion between the mandibular condyle and the glenoid fossa, with the disappearance of the joint space. (B) Both temporomandibular joints exhibit deformity and healing of the mandibular condyle displaced medially, along with extensive bony adhesions (red arrows).

A diagnosis of traumatic TMJ ankylosis was established, and in May 2021, bilateral GA with interpositional placement was performed under general anesthesia. On the right, an extraoral Al-Kayat-Bramley approach enabled the creation of a 10 mm gap, and a temporalis muscle flap was interposed (Figure 3A, 3B). On the left, an intraoral approach was used to create an 8 mm gap, with placement of a buccal fat pad flap (Figure 3C, 3D). Bilateral coronoidectomies were also performed to address persistent restriction in mandibular movement (Figure 4).

**Figure 3:**
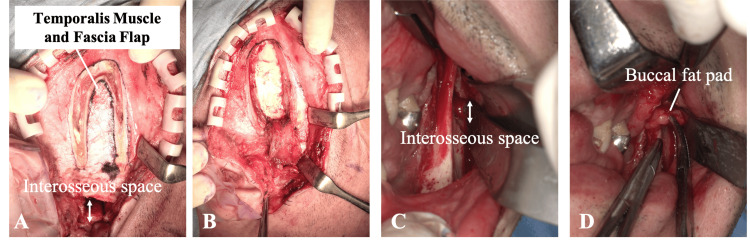
Intraoperative photographs. (A) A U-shaped incision measuring 80 × 25 mm is made, with its base at the inferior border of the temporalis fascia on the right side. (B) The temporalis fascia flap is folded downward and inserted into the interosseous space, and then sutured and fixed. (C) An osteotomy is performed from the mandibular notch to the posterior border of the mandibular ramus. (D) The buccal fat pad is dissected, inserted into the interosseous space, and sutured and fixed.

**Figure 4:**
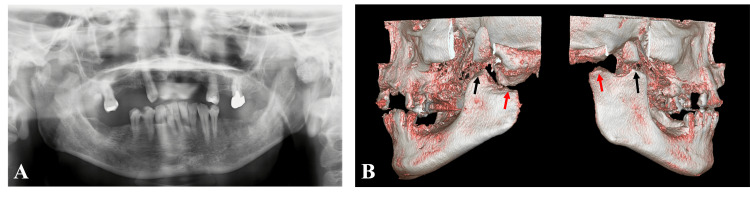
Postoperative panoramic X-ray images and cone-beam computed tomography 3D reconstruction images. (A) The panoramic X-ray image shows interosseous spaces at the bases of both condylar processes. (B) Bilateral coronoidectomy was performed (black arrows). On the right side, an osteotomy at the base of the condylar process created a gap (red arrow), with medial displacement of the superior bone fragment. On the left side, an osteotomy extending from the mandibular notch to the posterior border of the mandibular ramus resulted in the formation of a bony gap.

The operative time was approximately 74 minutes for the right side, and 94 minutes for the left side. Postoperative recovery was favorable. No sensory disturbance of the inferior alveolar nerve or the lingual nerve was observed on the left (intraoral) side, and no facial nerve palsy occurred. Mouth-opening exercises were initiated early. At discharge, the mouth opening was 43 mm, and at 3.5 years, it was 38 mm (Figure 5).

**Figure 5:**
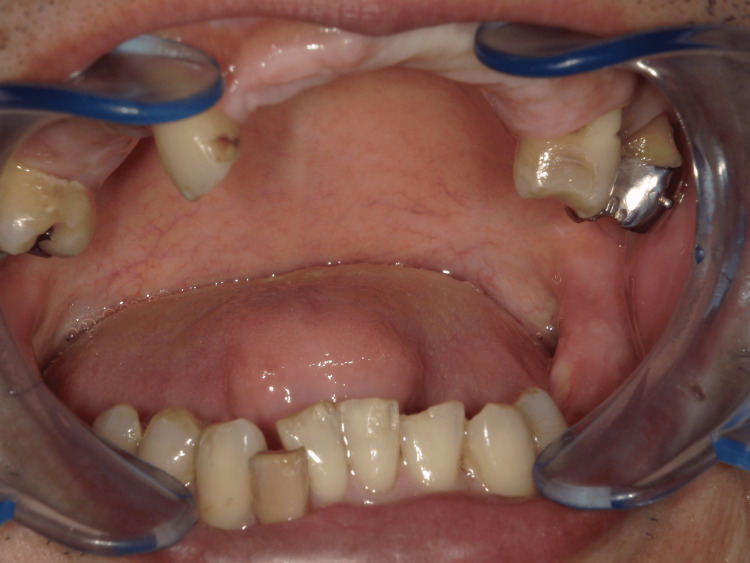
Intraoral photographs obtained at the time of discharge from our center. Postoperative photograph during mouth opening. The mouth opening is maintained at 38 mm.

The patient regained normal function and QOL.

## Discussion

TMJ ankylosis results from fibrous or bony adhesions between the condyle and the glenoid fossa, impairing mastication, hygiene, and speech, and often causing facial asymmetry. Diagnosis is straightforward in cases of bony ankylosis. Trauma is the leading cause (84.3%), followed by infection and arthritis [2]. Treatment generally involves surgical intervention, as advanced cases often require TMJ arthroplasty. The choice of treatment depends on the progression and etiology of the condition.

TMJ ankylosis restricts mouth opening and impairs mastication, markedly reducing QOL [1]. Traumatic TMJ ankylosis, particularly following bilateral condylar fractures, presents a complex therapeutic challenge [6]. Without appropriate treatment, it may lead to progressive joint dysfunction and jaw deformity [2].

GA is a common surgical strategy that restores movement by removing the ankylosed segment and creating an interarticular gap [3]. The extraoral approach provides wide access but risks facial nerve injury and scarring [4]. The intraoral approach is more aesthetic but has limited visibility and complexity [5].

We report a rare case of bilateral TMJ ankylosis following trauma, treated with GA using an extraoral approach on the right and an intraoral approach on the left. This report highlights the benefits of customizing surgical approaches for each joint.

In this case, an ultrasonic bone-cutting device was used to minimize damage to surrounding tissues while achieving efficient bone resection. Although a wider gap has generally been recommended to reduce the risk of recurrence, the osteotomy width on the left side was intentionally limited to 8 mm in the present case. Because the patient had already lost vision in the right eye, preservation of the minimization of surgical morbidity was prioritized. Therefore, an intraoral approach with conservative bone resection was selected on the left side, followed by intensive postoperative rehabilitation.

A temporalis fascia flap was used on the right side, while a fat tissue flap was employed on the left side. These flaps were interposed into the created bony gaps to prevent postoperative adhesions and restore joint function. For bony ankylosis, it is necessary to remove a bony adhesion with a width of 10-15 mm, as GA alone has been associated with a recurrence rate of 53% [7]. Therefore, IPG is often performed to prevent re-ankylosis, using autogenous materials such as temporalis muscle, dermal-fat grafts, or auricular cartilage. Although temporalis fascia flaps are flexible, well-vascularized, and stable, they may cause scarring and prolong surgery [8,9]. Conversely, fat tissue flaps are highly flexible and relatively easy to harvest but are prone to fibrosis under functional pressure and volume reduction due to resorption [10,11]. The choice of flap material should be carefully determined based on the patient’s anatomical conditions and the surgeon’s expertise.

Furthermore, surgical field approaches include extraoral and intraoral methods. Both have advantages and disadvantages, and the appropriate approach should be selected according to the specific case. Extraoral approaches allow for a wider surgical field and facilitate complex osteotomy procedures but are associated with risks such as facial nerve injury and postoperative scarring [12,13]. In contrast, intraoral approaches are superior from an aesthetic perspective and carry a lower risk of facial nerve injury; however, they are technically challenging and have limitations in terms of visibility and the use of interpositional materials [4,5]. Currently, there is no robust evidence indicating a substantial difference in recurrence rates between intraoral and extraoral approaches. Rather, recurrence appears to be more closely associated with the choice of surgical technique itself, including the completeness of the GA and the use of interpositional materials. Recent evidence suggests that the use of autogenous interpositional materials substantially reduces the risk of re-ankylosis compared with GA alone or alloplastic interpositions [14,15]. In the present case, given the extensive nature of the osseous fusion, the extraoral approach was selected to achieve optimal surgical access for both complete and radical resection of the bony ankylotic block and the reliable harvest and placement of the autogenous temporalis myofascial flap. This combined approach is regarded as the optimal strategy for minimizing the risk of recurrence and maximizing long-term mandibular function.

In the current case, the patient’s right eye prosthesis limited vision. To avoid left facial nerve injury and preserve vision, an intraoral approach was chosen for the left side. Accumulated evidence supports combining both approaches for optimal outcomes [16,17]. To our knowledge, no prior report has described the intentional use of bilateral but asymmetric approaches for GA in TMJ ankylosis. In this case, avoiding left facial nerve palsy was of paramount importance. Accordingly, an extraoral approach was used on the right side, and an intraoral approach on the left side, allowing us to tailor the surgical plan to the patient’s unique anatomical and functional needs. Such individualized selection of surgical approaches may contribute to more favorable postoperative outcomes, including improved stability and QOL.

Postoperative rehabilitation is considered essential for maintaining functional recovery through mouth opening exercises [5]. Early initiation of rehabilitation is recommended, ideally on the third postoperative day [4,12,18]. Physical therapy is indispensable for preventing recurrence [2]. Following established protocols, passive and active mouth opening exercises should be continued for 6-12 months to prevent re-ankylosis.

In this case, gradual mouth opening exercises using specialized equipment were implemented early in the postoperative period. Initial rehabilitation prioritized pain management and muscle stretching within a tolerable range. Physical therapy was also employed to improve the mobility of the masticatory muscles and TMJ. The exercises were continued for several months, with the patient performing them multiple times daily under guidance. These efforts prevented recurrence and improved joint function. Additionally, emphasis was placed on improving QOL, with a focus on mastication and speech abilities. The maximum mouth opening improved from 6 mm preoperatively to 43 mm immediately after surgery and was maintained at 38 mm after 3.5 years of follow-up. Although a slight reduction in mouth opening was observed during the follow-up period, this outcome was considered acceptable given the patient’s systemic sequelae after severe trauma and the associated limitations in physical rehabilitation. The patient maintained satisfactory occlusion and showed no clinical evidence of re-ankylosis.

This report has several limitations. As a single-case report, the findings cannot be generalized to all patients with TMJ ankylosis. Further studies with larger cohorts and longer follow-up are required to validate the effectiveness of individualized surgical approaches.

## Conclusions

In this case, bilateral TMJ ankylosis caused by trauma was successfully treated with GA using a temporalis fascia flap via an extraoral approach on the right side and a fat tissue flap via an intraoral approach on the left side. The postoperative course was favorable, with improvement in mouth opening capacity, maintenance of occlusion, and enhancement of QOL during the 3.5-year follow-up period. This case highlights the importance of individualized surgical planning based on patient-specific anatomical and functional considerations. However, as this report describes a single case, further studies are required to determine the broader applicability of this approach.
